# 1,2-Dimethyl-4,5-diphenyl­benzene determined on a Bruker SMART X2S benchtop crystallographic system

**DOI:** 10.1107/S1600536809015311

**Published:** 2009-04-30

**Authors:** Jonathan B. Briggs, Mikaël D. Jazdzyk, Glen P. Miller

**Affiliations:** a Department of Chemistry and Materials Science Program, University of New Hampshire, Durham, New Hampshire 03824-3598, USA

## Abstract

The title compound, C_20_H_18_, has two crystallographically independent mol­ecules in the asymmetric unit. The phenyl substituents of mol­ecule *A* are twisted away from the plane defined by the central benzene ring by 131.8 (2) and −52.7 (3)°. The phenyl substituents of mol­ecule *B* are twisted by −133.3 (2) and 50.9 (3)°. Each mol­ecule is stabilized by a pair of intra­molecular C(aryl, *sp* ^2^)—H⋯π inter­actions, as well as by several inter­molecular C(methyl, *sp* ^3^)—H⋯π inter­actions.

## Related literature

For potential applications and utility of the title compound as a synthetic inter­mediate, see: Kharasch *et al.* (1965 ▶); Horiuchi *et al.* (2008 ▶); Amine & Chen (2008 ▶); Eaton (2008 ▶); Peters & Friedrichsen (1995 ▶); Segura & Martín (1999 ▶). For the synthesis and related crystal structures, see: Maier *et al.*, (1969 ▶); Maeyama & Yonezawa (2003 ▶); Brown & Levy (1979 ▶).
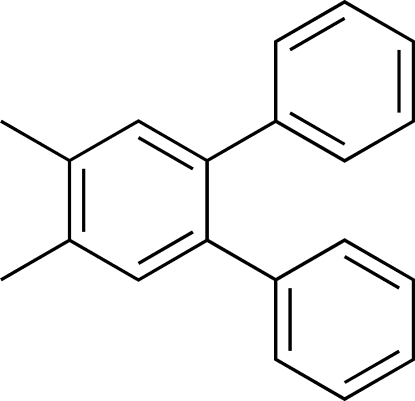


## Experimental

### 

#### Crystal data

C_20_H_18_
*M* *_r_* = 258.34
Triclinic, 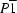

*a* = 9.3033 (7) Å
*b* = 10.7546 (9) Å
*c* = 16.3322 (12) Å
α = 93.793 (3)°
β = 98.934 (3)°
γ = 106.549 (2)°
*V* = 1536.8 (2) Å^3^
*Z* = 4
Mo *K*α radiation
μ = 0.06 mm^−1^
*T* = 296 K
0.50 × 0.50 × 0.05 mm

#### Data collection

Bruker SMART X2S diffractometer
Absorption correction: multi-scan (*SADABS*; Bruker, 2007 ▶) *T*_min_ = 0.969, *T*_max_ = 0.997
15460 measured reflections
5450 independent reflections
3881 reflections with *I* > 2σ(*I*)
*R*_int_ = 0.031

#### Refinement

*R*[*F* ^2^ > 2σ(*F* ^2^)] = 0.046
*wR*(*F* ^2^) = 0.166
*S* = 0.87
5450 reflections
365 parameters
H-atom parameters constrained
Δρ_max_ = 0.25 e Å^−3^
Δρ_min_ = −0.32 e Å^−3^

### 

Data collection: *GIS* (Bruker, 2007 ▶); cell refinement: *SAINT* (Bruker, 2007 ▶); data reduction: *SAINT*; program(s) used to solve structure: *SHELXS97* (Sheldrick, 2008 ▶); program(s) used to refine structure: *SHELXL97* (Sheldrick, 2008 ▶); molecular graphics: *SHELXTL* (Sheldrick, 2008 ▶); software used to prepare material for publication: *SHELXTL*.

## Supplementary Material

Crystal structure: contains datablocks I, global. DOI: 10.1107/S1600536809015311/fl2245sup1.cif

Structure factors: contains datablocks I. DOI: 10.1107/S1600536809015311/fl2245Isup2.hkl

Additional supplementary materials:  crystallographic information; 3D view; checkCIF report

## Figures and Tables

**Table 1:** C—H⋯π interaction geometry (Å, °)

C—H⋯π	C—H	H⋯π	C⋯π	C—H⋯π
C7*A*—H7*A*2⋯C5*A*	0.96	2.892	3.780	154.41
C7*A*—H7*A*3⋯C12*A*	0.96	2.920	3.824	157.38
C8*A*—H8*A*1⋯C16*B*	0.96	3.014	3.924	158.82
C14*A*—H14*A*⋯C15*A*	0.93	2.811	3.126	101.09
C16*A*—H16*A*⋯C9*A*	0.93	2.881	3.167	99.24
C7*B*—H7*B*1⋯C11*B*	0.96	2.991	3.666	128.53
C8*B*—H8*B*1⋯C12*B*	0.96	2.943	3.809	150.67
C14*B*—H14*B*⋯C15*B*	0.93	2.784	3.129	103.11
C16*B*—H16*B*⋯C9*B*	0.93	2.823	3.138	101.13

**Fig. 1.**
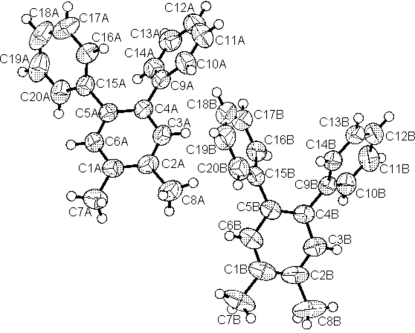
The molecular structure showing the crystallographic labelling scheme and displacement ellipsoids drawn at the 50% probability level. H atoms are shown as spheres of arbitrary radii.

**Fig. 2.**
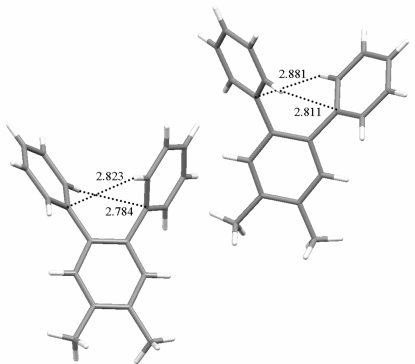
Perspective view of the title molecule showing the two pairs of stabilizing intramolecular C(aryl, sp2)-H···π interactions involving one ortho hydrogen atom on each phenyl substituent and one π bond associated with the ipso carbon of the other phenyl substituent.

**Fig. 3.**
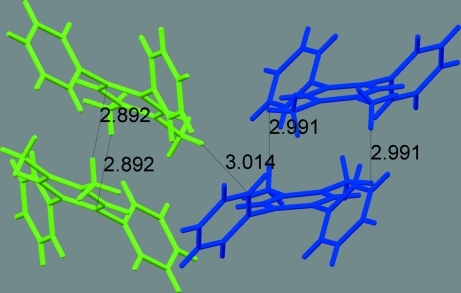
Perspective view of the title molecule showing stabilizing intermolecular C(methyl, sp3)-H···π interactions involving methyl substituents and π bonds associated with carbons of neighboring phenyl substituents. Molecule A is colored in green and molecule B is colored in blue. One unique type A—B as well as one type A—A and one type B—B CH···π interaction is shown.

**Fig. 4.**
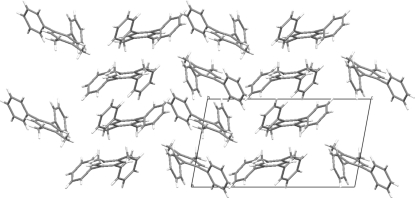
Perspective view of long range packing in the crystal structure.

**Fig. 5.**
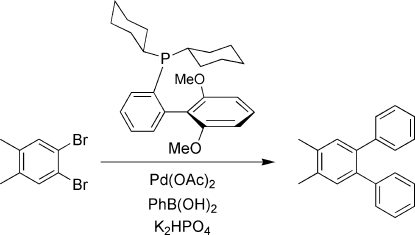
Synthesis of the title compound, 1,2-dimethyl-4,5-diphenylbenzene.

## supplementary crystallographic information

### Comment

*o-*Terphenyl has been utilized as a photochemical precursor to triphenylene (Kharasch *et al.*, (1965)), as part of a cathode active material layer in battery applications (Horiuchi *et al.*, (2008)), as a stabilizing additive in non-aqueous electrolytes (Amine & Chen, (2008)), and as a voltage stabilizer within the insulating layer of power cables (Eaton, (2008)). The title compound, an *o-*terphenyl derivative, is a potentially interesting synthetic intermediate leading to novel isobenzofuran (Peters & Friedrichsen, (1995)) and/or quinodimethane (Segura & Martín, (1999)) species and was first prepared in 1969 (Maier *et al.*, (1969)). The synthesis of *o-*terphenyl derivatives was recently reviewed (Maeyama & Yonezawa, (2003)). A crystal structure of unsubstituted *o-*terphenyl has been published (Brown & Levy, (1979)).

The asymmetric unit of (I) contains two molecules (Fig. 1). The relative rotations of the phenyl substituents at C4 and C5 are influenced by a pair of stabilizing intramolecular *C*(aryl, *sp^2^*)-H···π interactions involving one *ortho* hydrogen atom on each phenyl substituent and one π bond associated with the *ipso* carbon of the other phenyl substituent (Fig. 2). The atoms of closest contact (H*_ortho_*—C*_ipso_*) are separated by 2.784 Å (Table 1). An *MM2* force field minimization for a single molecule in a vacuum places the same two atoms 2.80 Å apart indicating that the molecular conformation within the crystal lattice is little influenced by packing forces. There are, *e.g.*, no significant π-π interactions in the crystal structure.

Weaker intermolecular CH···π interactions involving both methyl substituents and π bonds on adjacent molecules help to define the spacing between molecules in the crystal structure (Table 1, Figs. 3–4). There are a total of five unique intermolecular CH···π interactions. Within the asymmetric unit, there is one intermolecular CH···π interaction (H8A1*_methyl_*···C16B*_ortho_*, 3.014 Å) involving one molecule A and one molecule B (A—B). Additionally, each molecule A and molecule B within the asymmetric unit has two unique CH···π interactions involving other molecules of the same type (2 A—A; 2 B—B). The two type A—A intermolecular CH···π interactions can be described as H7A2*_methyl_*-C5A*_central ring_* (2.892 Å) and H7A3*_methyl_*-C12A*_para_* (2.920 Å). The two type B—B intermolecular CH···π interactions can be described as H7B1*_methyl_*-C11B*_meta_* (2.991 Å) and H8B1*_methyl_*-C12B*_para_* (2.943 Å). Figure 3 illustrates the one unique type A—B intermolecular CH···π interaction as well as one type A—A and one type B—B CH···π interaction.

### Experimental

The title compound was prepared as illustrated in Fig. 5. An oven-dried glass pressure vessel containing a magnetic stir bar was charged with palladium(II) acetate (0.034 g, 0.152 mmol), 1,2-dibromo-4,5-dimethylbenzene (2 g, 7.58 mmol), 2-dicyclohexylphosphino-2',6'-dimethoxybiphenyl (0.124 g, 0.303 mmol), phenylboronic acid (2.77 g, 22.7 mmol) and powdered, anhydrous potassium phosphate (11.26 g, 53.0 mmol). Dry THF (20 ml) was added and N_2_ gas was bubbled through the resulting mixture for 15 min. The glass pressure vessel was sealed with a Teflon cap and heated at 75 °C for 20 h with stirring. The reaction mixture was allowed to cool to room temperature after which the mixture was diluted with diethyl ether (30 ml) and washed with water three times. The organic layer was dried over magnesium sulfate and concentrated at reduced pressure. The crude product was purified by flash column chromatography on silica gel using hexane-chloroform (80/20 *v/v*) as eluent. The title compound, 1,2-dimethyl-4,5-diphenylbenzene, was obtained in 76% isolated yield. ^1^H NMR (400 MHz, CDCl_3_) δ 2.35 (s, 6H), 7.11–7.21 (m, 12H); ^13^C NMR (100 MHz, CDCl_3_) δ 19.6 (CH3), 126.4 (CH), 128.0 (CH), 130.1 (CH), 132.1 (CH), 136.0 (C), 138.2 (C), 141.7 (C). An X-ray grade crystal was obtained by slow evaporation of a dichloromethane solution.

### Crystal data

**Table:**

C_20_H_18_	*Z* = 4
*M**_r_* = 258.34	*F*(000) = 552
Triclinic, *P*1	*D*_x_ = 1.117 Mg m^−^^3^
Hall symbol: -P 1	Mo *K*α radiation, λ = 0.71073 Å
*a* = 9.3033 (7) Å	Cell parameters from 5281 reflections
*b* = 10.7546 (9) Å	θ = 1.3–25.2°
*c* = 16.3322 (12) Å	µ = 0.06 mm^−^^1^
α = 93.793 (3)°	*T* = 296 K
β = 98.934 (3)°	Plate, clear colourless
γ = 106.549 (2)°	0.50 × 0.50 × 0.05 mm
*V* = 1536.8 (2) Å^3^	

### Data collection

**Table:**

Bruker SMART X2S diffractometer	5450 independent reflections
Radiation source: micro-focus sealed tube	3881 reflections with *I* > 2σ(*I*)
doubly curved silicon crystal	*R*_int_ = 0.031
ω scans	θ_max_ = 25.2°, θ_min_ = 1.3°
Absorption correction: multi-scan (*SADABS*; Bruker, 2007)	*h* = −11→11
*T*_min_ = 0.969, *T*_max_ = 0.997	*k* = −12→12
15460 measured reflections	*l* = −19→19

### Refinement

**Table:**

Refinement on *F*^2^	Primary atom site location: structure-invariant direct methods
Least-squares matrix: full	Secondary atom site location: difference Fourier map
*R*[*F*^2^ > 2σ(*F*^2^)] = 0.046	Hydrogen site location: inferred from neighbouring sites
*wR*(*F*^2^) = 0.166	H-atom parameters constrained
*S* = 0.87	*w* = 1/[σ^2^(*F*_o_^2^) + (0.1*P*)^2^ + 0.5*P*] where *P* = (*F*_o_^2^ + 2*F*_c_^2^)/3
5450 reflections	(Δ/σ)_max_ < 0.001
365 parameters	Δρ_max_ = 0.25 e Å^−^^3^
0 restraints	Δρ_min_ = −0.32 e Å^−^^3^

### Special details

**Table:**

Geometry. All e.s.d.'s (except the e.s.d. in the dihedral angle between two l.s. planes) are estimated using the full covariance matrix. The cell e.s.d.'s are taken into account individually in the estimation of e.s.d.'s in distances, angles and torsion angles; correlations between e.s.d.'s in cell parameters are only used when they are defined by crystal symmetry. An approximate (isotropic) treatment of cell e.s.d.'s is used for estimating e.s.d.'s involving l.s. planes.
Refinement. Refinement of *F*^2^ against ALL reflections. The weighted *R*-factor *wR* and goodness of fit *S* are based on *F*^2^, conventional *R*-factors *R* are based on *F*, with *F* set to zero for negative *F*^2^. The threshold expression of *F*^2^ > σ(*F*^2^) is used only for calculating *R*-factors(gt) *etc*. and is not relevant to the choice of reflections for refinement. *R*-factors based on *F*^2^ are statistically about twice as large as those based on *F*, and *R*- factors based on ALL data will be even larger.

### Fractional atomic coordinates and isotropic or equivalent isotropic displacement parameters (Å^2^)

**Table:**

	*x*	*y*	*z*	*U*_iso_*/*U*_eq_	
C1A	0.6791 (2)	1.08007 (18)	0.97275 (13)	0.0567 (5)	
C2A	0.6269 (2)	1.01769 (19)	0.89127 (13)	0.0602 (5)	
C3A	0.6488 (2)	0.89721 (19)	0.87258 (12)	0.0595 (5)	
H3A	0.6152	0.8566	0.8181	0.071*	
C4A	0.7186 (2)	0.83381 (17)	0.93120 (11)	0.0527 (4)	
C5A	0.7660 (2)	0.89453 (17)	1.01371 (11)	0.0507 (4)	
C6A	0.7463 (2)	1.01665 (18)	1.03178 (12)	0.0555 (5)	
H6A	0.7800	1.0577	1.0862	0.067*	
C7A	0.6614 (3)	1.2118 (2)	0.99848 (16)	0.0750 (6)	
H7A1	0.6947	1.2338	1.0577	0.112*	
H7A2	0.5562	1.2084	0.9840	0.112*	
H7A3	0.7221	1.2768	0.9701	0.112*	
C8A	0.5478 (3)	1.0778 (2)	0.82374 (16)	0.0822 (7)	
H8A1	0.5130	1.0175	0.7737	0.123*	
H8A2	0.6180	1.1570	0.8129	0.123*	
H8A3	0.4623	1.0970	0.8418	0.123*	
C9A	0.7423 (2)	0.70697 (18)	0.90456 (11)	0.0547 (5)	
C10A	0.6240 (3)	0.6064 (2)	0.85743 (14)	0.0714 (6)	
H10A	0.5287	0.6184	0.8425	0.086*	
C11A	0.6455 (3)	0.4888 (2)	0.83227 (16)	0.0858 (7)	
H11A	0.5645	0.4221	0.8013	0.103*	
C12A	0.7859 (3)	0.4703 (2)	0.85288 (15)	0.0788 (7)	
H12A	0.8006	0.3915	0.8352	0.095*	
C13A	0.9046 (3)	0.5682 (2)	0.89956 (14)	0.0703 (6)	
H13A	0.9996	0.5554	0.9142	0.084*	
C14A	0.8836 (2)	0.6858 (2)	0.92493 (12)	0.0610 (5)	
H14A	0.9652	0.7519	0.9561	0.073*	
C15A	0.8283 (2)	0.83145 (19)	1.08364 (11)	0.0543 (5)	
C16A	0.7485 (3)	0.7074 (2)	1.09899 (14)	0.0686 (6)	
H16A	0.6561	0.6625	1.0646	0.082*	
C17A	0.8049 (3)	0.6501 (3)	1.16471 (17)	0.0899 (8)	
H17A	0.7512	0.5667	1.1743	0.108*	
C18A	0.9418 (4)	0.7172 (4)	1.21634 (16)	0.0999 (10)	
H18A	0.9797	0.6791	1.2610	0.120*	
C19A	1.0214 (3)	0.8393 (3)	1.20186 (15)	0.0925 (8)	
H19A	1.1137	0.8837	1.2365	0.111*	
C20A	0.9653 (3)	0.8970 (2)	1.13604 (13)	0.0712 (6)	
H20A	1.0197	0.9803	1.1269	0.085*	
C1B	0.2893 (2)	1.2264 (2)	0.56098 (18)	0.0736 (6)	
C2B	0.3057 (3)	1.2009 (2)	0.47854 (18)	0.0767 (7)	
C3B	0.2838 (2)	1.0722 (2)	0.44640 (15)	0.0670 (6)	
H3B	0.2929	1.0554	0.3912	0.080*	
C4B	0.2488 (2)	0.96758 (17)	0.49331 (12)	0.0534 (4)	
C5B	0.2360 (2)	0.99418 (17)	0.57707 (13)	0.0549 (5)	
C6B	0.2545 (2)	1.12292 (19)	0.60791 (15)	0.0670 (6)	
H6B	0.2429	1.1400	0.6627	0.080*	
C7B	0.3143 (3)	1.3648 (2)	0.6001 (2)	0.1030 (10)	
H7B1	0.4207	1.4124	0.6084	0.155*	
H7B2	0.2808	1.3622	0.6528	0.155*	
H7B3	0.2570	1.4073	0.5637	0.155*	
C8B	0.3495 (4)	1.3080 (3)	0.4225 (2)	0.1211 (12)	
H8B1	0.2807	1.3598	0.4215	0.182*	
H8B2	0.3436	1.2692	0.3670	0.182*	
H8B3	0.4517	1.3624	0.4437	0.182*	
C9B	0.2263 (2)	0.83396 (17)	0.45237 (11)	0.0508 (4)	
C10B	0.3309 (2)	0.8121 (2)	0.40513 (13)	0.0646 (5)	
H10B	0.4156	0.8809	0.4008	0.077*	
C11B	0.3109 (3)	0.6904 (3)	0.36479 (15)	0.0773 (6)	
H11B	0.3815	0.6779	0.3331	0.093*	
C12B	0.1874 (3)	0.5871 (2)	0.37099 (14)	0.0756 (7)	
H12B	0.1748	0.5049	0.3438	0.091*	
C13B	0.0818 (3)	0.6059 (2)	0.41787 (13)	0.0683 (6)	
H13B	−0.0017	0.5363	0.4225	0.082*	
C14B	0.1012 (2)	0.72904 (18)	0.45784 (12)	0.0586 (5)	
H14B	0.0295	0.7416	0.4887	0.070*	
C15B	0.2085 (2)	0.89099 (18)	0.63430 (12)	0.0569 (5)	
C16B	0.2983 (3)	0.8077 (2)	0.64348 (13)	0.0652 (5)	
H16B	0.3765	0.8157	0.6131	0.078*	
C17B	0.2728 (3)	0.7128 (2)	0.69743 (15)	0.0832 (7)	
H17B	0.3336	0.6575	0.7028	0.100*	
C18B	0.1585 (4)	0.7000 (3)	0.74299 (16)	0.0958 (9)	
H18B	0.1418	0.6360	0.7790	0.115*	
C19B	0.0694 (4)	0.7813 (3)	0.73542 (16)	0.0950 (9)	
H19B	−0.0081	0.7725	0.7663	0.114*	
C20B	0.0939 (3)	0.8768 (2)	0.68189 (14)	0.0762 (6)	
H20B	0.0330	0.9321	0.6776	0.091*	

### Atomic displacement parameters (Å^2^)

**Table:**

	*U*^11^	*U*^22^	*U*^33^	*U*^12^	*U*^13^	*U*^23^
C1A	0.0489 (10)	0.0504 (10)	0.0671 (12)	0.0102 (8)	0.0060 (9)	0.0126 (9)
C2A	0.0538 (11)	0.0589 (11)	0.0632 (12)	0.0116 (9)	0.0017 (9)	0.0185 (9)
C3A	0.0609 (12)	0.0608 (12)	0.0490 (10)	0.0114 (9)	−0.0011 (9)	0.0083 (9)
C4A	0.0492 (10)	0.0530 (10)	0.0511 (10)	0.0094 (8)	0.0043 (8)	0.0095 (8)
C5A	0.0452 (9)	0.0532 (10)	0.0506 (10)	0.0110 (8)	0.0045 (8)	0.0102 (8)
C6A	0.0526 (10)	0.0544 (11)	0.0526 (10)	0.0096 (8)	0.0024 (8)	0.0044 (8)
C7A	0.0728 (14)	0.0597 (13)	0.0910 (16)	0.0206 (11)	0.0075 (12)	0.0120 (11)
C8A	0.0858 (16)	0.0770 (15)	0.0801 (15)	0.0267 (13)	−0.0071 (13)	0.0244 (12)
C9A	0.0594 (11)	0.0552 (11)	0.0475 (10)	0.0132 (9)	0.0098 (8)	0.0100 (8)
C10A	0.0699 (13)	0.0681 (13)	0.0691 (13)	0.0176 (11)	0.0023 (11)	−0.0036 (11)
C11A	0.0900 (18)	0.0720 (15)	0.0832 (16)	0.0149 (13)	0.0075 (13)	−0.0150 (12)
C12A	0.1027 (19)	0.0640 (14)	0.0766 (15)	0.0291 (13)	0.0306 (14)	0.0039 (11)
C13A	0.0769 (14)	0.0765 (14)	0.0696 (13)	0.0319 (12)	0.0289 (12)	0.0176 (11)
C14A	0.0615 (12)	0.0629 (12)	0.0588 (11)	0.0156 (9)	0.0157 (9)	0.0114 (9)
C15A	0.0542 (11)	0.0673 (12)	0.0469 (10)	0.0264 (9)	0.0088 (8)	0.0091 (9)
C16A	0.0686 (13)	0.0759 (14)	0.0676 (13)	0.0271 (11)	0.0139 (10)	0.0252 (11)
C17A	0.101 (2)	0.107 (2)	0.0881 (18)	0.0555 (17)	0.0322 (16)	0.0513 (16)
C18A	0.113 (2)	0.161 (3)	0.0649 (15)	0.090 (2)	0.0245 (16)	0.0473 (18)
C19A	0.0822 (17)	0.145 (3)	0.0585 (14)	0.0564 (18)	−0.0049 (12)	0.0087 (16)
C20A	0.0644 (13)	0.0904 (16)	0.0576 (12)	0.0285 (12)	−0.0011 (10)	0.0046 (11)
C1B	0.0521 (12)	0.0495 (11)	0.119 (2)	0.0181 (9)	0.0116 (12)	0.0077 (12)
C2B	0.0601 (13)	0.0559 (12)	0.115 (2)	0.0187 (10)	0.0071 (13)	0.0307 (13)
C3B	0.0625 (12)	0.0623 (13)	0.0765 (14)	0.0185 (10)	0.0085 (10)	0.0205 (10)
C4B	0.0445 (10)	0.0502 (10)	0.0649 (12)	0.0148 (8)	0.0052 (8)	0.0111 (9)
C5B	0.0465 (10)	0.0493 (10)	0.0678 (12)	0.0137 (8)	0.0096 (9)	0.0032 (9)
C6B	0.0566 (12)	0.0540 (12)	0.0895 (15)	0.0153 (9)	0.0173 (11)	−0.0022 (11)
C7B	0.0788 (16)	0.0497 (13)	0.178 (3)	0.0202 (12)	0.0189 (18)	−0.0019 (15)
C8B	0.133 (3)	0.0742 (17)	0.164 (3)	0.0323 (17)	0.028 (2)	0.0610 (19)
C9B	0.0499 (10)	0.0542 (10)	0.0473 (10)	0.0179 (8)	0.0008 (8)	0.0089 (8)
C10B	0.0574 (12)	0.0755 (14)	0.0597 (12)	0.0216 (10)	0.0056 (9)	0.0042 (10)
C11B	0.0712 (15)	0.0945 (17)	0.0694 (14)	0.0399 (14)	0.0012 (11)	−0.0092 (12)
C12B	0.0977 (18)	0.0681 (14)	0.0625 (13)	0.0447 (14)	−0.0140 (12)	−0.0042 (11)
C13B	0.0821 (15)	0.0544 (12)	0.0576 (12)	0.0139 (10)	−0.0080 (11)	0.0087 (9)
C14B	0.0613 (12)	0.0579 (11)	0.0529 (11)	0.0146 (9)	0.0049 (9)	0.0074 (9)
C15B	0.0603 (11)	0.0493 (10)	0.0524 (10)	0.0073 (9)	0.0053 (9)	−0.0031 (8)
C16B	0.0709 (13)	0.0606 (12)	0.0592 (12)	0.0185 (10)	0.0007 (10)	0.0040 (10)
C17B	0.109 (2)	0.0675 (14)	0.0639 (14)	0.0247 (13)	−0.0077 (14)	0.0088 (11)
C18B	0.134 (3)	0.0789 (17)	0.0597 (14)	0.0105 (17)	0.0105 (16)	0.0154 (12)
C19B	0.115 (2)	0.0932 (19)	0.0661 (15)	0.0049 (17)	0.0355 (15)	0.0097 (14)
C20B	0.0830 (15)	0.0723 (14)	0.0707 (14)	0.0155 (12)	0.0243 (12)	−0.0002 (11)

### Geometric parameters (Å, °)

**Table:**

C1A—C6A	1.389 (3)	C1B—C6B	1.384 (3)
C1A—C2A	1.397 (3)	C1B—C2B	1.397 (4)
C1A—C7A	1.512 (3)	C1B—C7B	1.519 (3)
C2A—C3A	1.390 (3)	C2B—C3B	1.394 (3)
C2A—C8A	1.513 (3)	C2B—C8B	1.522 (3)
C3A—C4A	1.396 (3)	C3B—C4B	1.394 (3)
C3A—H3A	0.9300	C3B—H3B	0.9300
C4A—C5A	1.404 (3)	C4B—C5B	1.409 (3)
C4A—C9A	1.491 (3)	C4B—C9B	1.488 (3)
C5A—C6A	1.396 (3)	C5B—C6B	1.395 (3)
C5A—C15A	1.491 (3)	C5B—C15B	1.490 (3)
C6A—H6A	0.9300	C6B—H6B	0.9300
C7A—H7A1	0.9600	C7B—H7B1	0.9600
C7A—H7A2	0.9600	C7B—H7B2	0.9600
C7A—H7A3	0.9600	C7B—H7B3	0.9600
C8A—H8A1	0.9600	C8B—H8B1	0.9600
C8A—H8A2	0.9600	C8B—H8B2	0.9600
C8A—H8A3	0.9600	C8B—H8B3	0.9600
C9A—C10A	1.386 (3)	C9B—C14B	1.391 (3)
C9A—C14A	1.391 (3)	C9B—C10B	1.391 (3)
C10A—C11A	1.382 (3)	C10B—C11B	1.375 (3)
C10A—H10A	0.9300	C10B—H10B	0.9300
C11A—C12A	1.371 (4)	C11B—C12B	1.374 (3)
C11A—H11A	0.9300	C11B—H11B	0.9300
C12A—C13A	1.371 (3)	C12B—C13B	1.385 (3)
C12A—H12A	0.9300	C12B—H12B	0.9300
C13A—C14A	1.381 (3)	C13B—C14B	1.388 (3)
C13A—H13A	0.9300	C13B—H13B	0.9300
C14A—H14A	0.9300	C14B—H14B	0.9300
C15A—C20A	1.386 (3)	C15B—C16B	1.389 (3)
C15A—C16A	1.390 (3)	C15B—C20B	1.394 (3)
C16A—C17A	1.380 (3)	C16B—C17B	1.386 (3)
C16A—H16A	0.9300	C16B—H16B	0.9300
C17A—C18A	1.384 (4)	C17B—C18B	1.371 (4)
C17A—H17A	0.9300	C17B—H17B	0.9300
C18A—C19A	1.367 (4)	C18B—C19B	1.364 (4)
C18A—H18A	0.9300	C18B—H18B	0.9300
C19A—C20A	1.383 (3)	C19B—C20B	1.385 (3)
C19A—H19A	0.9300	C19B—H19B	0.9300
C20A—H20A	0.9300	C20B—H20B	0.9300
			
C6A—C1A—C2A	118.28 (18)	C6B—C1B—C2B	118.5 (2)
C6A—C1A—C7A	119.56 (18)	C6B—C1B—C7B	120.1 (3)
C2A—C1A—C7A	122.15 (18)	C2B—C1B—C7B	121.3 (2)
C3A—C2A—C1A	118.55 (17)	C3B—C2B—C1B	118.8 (2)
C3A—C2A—C8A	119.69 (19)	C3B—C2B—C8B	118.4 (3)
C1A—C2A—C8A	121.76 (19)	C1B—C2B—C8B	122.8 (2)
C2A—C3A—C4A	123.52 (18)	C2B—C3B—C4B	123.0 (2)
C2A—C3A—H3A	118.2	C2B—C3B—H3B	118.5
C4A—C3A—H3A	118.2	C4B—C3B—H3B	118.5
C3A—C4A—C5A	117.80 (17)	C3B—C4B—C5B	118.06 (18)
C3A—C4A—C9A	119.66 (17)	C3B—C4B—C9B	118.42 (18)
C5A—C4A—C9A	122.53 (16)	C5B—C4B—C9B	123.52 (16)
C6A—C5A—C4A	118.40 (16)	C6B—C5B—C4B	118.42 (18)
C6A—C5A—C15A	118.50 (16)	C6B—C5B—C15B	119.01 (18)
C4A—C5A—C15A	123.02 (16)	C4B—C5B—C15B	122.54 (16)
C1A—C6A—C5A	123.39 (18)	C1B—C6B—C5B	123.2 (2)
C1A—C6A—H6A	118.3	C1B—C6B—H6B	118.4
C5A—C6A—H6A	118.3	C5B—C6B—H6B	118.4
C1A—C7A—H7A1	109.5	C1B—C7B—H7B1	109.5
C1A—C7A—H7A2	109.5	C1B—C7B—H7B2	109.5
H7A1—C7A—H7A2	109.5	H7B1—C7B—H7B2	109.5
C1A—C7A—H7A3	109.5	C1B—C7B—H7B3	109.5
H7A1—C7A—H7A3	109.5	H7B1—C7B—H7B3	109.5
H7A2—C7A—H7A3	109.5	H7B2—C7B—H7B3	109.5
C2A—C8A—H8A1	109.5	C2B—C8B—H8B1	109.5
C2A—C8A—H8A2	109.5	C2B—C8B—H8B2	109.5
H8A1—C8A—H8A2	109.5	H8B1—C8B—H8B2	109.5
C2A—C8A—H8A3	109.5	C2B—C8B—H8B3	109.5
H8A1—C8A—H8A3	109.5	H8B1—C8B—H8B3	109.5
H8A2—C8A—H8A3	109.5	H8B2—C8B—H8B3	109.5
C10A—C9A—C14A	117.72 (19)	C14B—C9B—C10B	118.09 (18)
C10A—C9A—C4A	120.83 (18)	C14B—C9B—C4B	122.16 (17)
C14A—C9A—C4A	121.45 (17)	C10B—C9B—C4B	119.72 (17)
C11A—C10A—C9A	121.1 (2)	C11B—C10B—C9B	121.0 (2)
C11A—C10A—H10A	119.5	C11B—C10B—H10B	119.5
C9A—C10A—H10A	119.5	C9B—C10B—H10B	119.5
C12A—C11A—C10A	120.2 (2)	C10B—C11B—C12B	120.5 (2)
C12A—C11A—H11A	119.9	C10B—C11B—H11B	119.7
C10A—C11A—H11A	119.9	C12B—C11B—H11B	119.7
C13A—C12A—C11A	119.8 (2)	C11B—C12B—C13B	119.7 (2)
C13A—C12A—H12A	120.1	C11B—C12B—H12B	120.1
C11A—C12A—H12A	120.1	C13B—C12B—H12B	120.1
C12A—C13A—C14A	120.2 (2)	C12B—C13B—C14B	119.7 (2)
C12A—C13A—H13A	119.9	C12B—C13B—H13B	120.1
C14A—C13A—H13A	119.9	C14B—C13B—H13B	120.1
C13A—C14A—C9A	121.0 (2)	C13B—C14B—C9B	120.9 (2)
C13A—C14A—H14A	119.5	C13B—C14B—H14B	119.5
C9A—C14A—H14A	119.5	C9B—C14B—H14B	119.5
C20A—C15A—C16A	118.71 (19)	C16B—C15B—C20B	117.8 (2)
C20A—C15A—C5A	120.54 (18)	C16B—C15B—C5B	121.12 (18)
C16A—C15A—C5A	120.72 (18)	C20B—C15B—C5B	121.11 (19)
C17A—C16A—C15A	120.7 (2)	C17B—C16B—C15B	120.7 (2)
C17A—C16A—H16A	119.7	C17B—C16B—H16B	119.6
C15A—C16A—H16A	119.7	C15B—C16B—H16B	119.6
C16A—C17A—C18A	119.7 (3)	C18B—C17B—C16B	120.4 (3)
C16A—C17A—H17A	120.1	C18B—C17B—H17B	119.8
C18A—C17A—H17A	120.1	C16B—C17B—H17B	119.8
C19A—C18A—C17A	120.1 (2)	C19B—C18B—C17B	119.9 (3)
C19A—C18A—H18A	119.9	C19B—C18B—H18B	120.0
C17A—C18A—H18A	119.9	C17B—C18B—H18B	120.0
C18A—C19A—C20A	120.3 (3)	C18B—C19B—C20B	120.3 (3)
C18A—C19A—H19A	119.8	C18B—C19B—H19B	119.8
C20A—C19A—H19A	119.8	C20B—C19B—H19B	119.8
C19A—C20A—C15A	120.4 (2)	C19B—C20B—C15B	120.9 (2)
C19A—C20A—H20A	119.8	C19B—C20B—H20B	119.6
C15A—C20A—H20A	119.8	C15B—C20B—H20B	119.6
			
C6A—C1A—C2A—C3A	−1.9 (3)	C6B—C1B—C2B—C3B	1.2 (3)
C7A—C1A—C2A—C3A	179.46 (18)	C7B—C1B—C2B—C3B	179.0 (2)
C6A—C1A—C2A—C8A	178.02 (19)	C6B—C1B—C2B—C8B	−177.7 (2)
C7A—C1A—C2A—C8A	−0.6 (3)	C7B—C1B—C2B—C8B	0.1 (4)
C1A—C2A—C3A—C4A	0.9 (3)	C1B—C2B—C3B—C4B	−1.2 (3)
C8A—C2A—C3A—C4A	−179.04 (19)	C8B—C2B—C3B—C4B	177.7 (2)
C2A—C3A—C4A—C5A	1.3 (3)	C2B—C3B—C4B—C5B	−0.3 (3)
C2A—C3A—C4A—C9A	−177.72 (18)	C2B—C3B—C4B—C9B	179.45 (18)
C3A—C4A—C5A—C6A	−2.5 (3)	C3B—C4B—C5B—C6B	1.9 (3)
C9A—C4A—C5A—C6A	176.55 (16)	C9B—C4B—C5B—C6B	−177.88 (17)
C3A—C4A—C5A—C15A	174.07 (17)	C3B—C4B—C5B—C15B	−176.16 (18)
C9A—C4A—C5A—C15A	−6.9 (3)	C9B—C4B—C5B—C15B	4.1 (3)
C2A—C1A—C6A—C5A	0.7 (3)	C2B—C1B—C6B—C5B	0.5 (3)
C7A—C1A—C6A—C5A	179.40 (18)	C7B—C1B—C6B—C5B	−177.4 (2)
C4A—C5A—C6A—C1A	1.5 (3)	C4B—C5B—C6B—C1B	−2.0 (3)
C15A—C5A—C6A—C1A	−175.20 (17)	C15B—C5B—C6B—C1B	176.11 (19)
C3A—C4A—C9A—C10A	−49.2 (3)	C3B—C4B—C9B—C14B	−131.5 (2)
C5A—C4A—C9A—C10A	131.8 (2)	C5B—C4B—C9B—C14B	48.3 (3)
C3A—C4A—C9A—C14A	129.9 (2)	C3B—C4B—C9B—C10B	46.9 (2)
C5A—C4A—C9A—C14A	−49.1 (3)	C5B—C4B—C9B—C10B	−133.3 (2)
C14A—C9A—C10A—C11A	0.6 (3)	C14B—C9B—C10B—C11B	0.2 (3)
C4A—C9A—C10A—C11A	179.7 (2)	C4B—C9B—C10B—C11B	−178.27 (18)
C9A—C10A—C11A—C12A	−0.8 (4)	C9B—C10B—C11B—C12B	−0.6 (3)
C10A—C11A—C12A—C13A	0.9 (4)	C10B—C11B—C12B—C13B	0.4 (3)
C11A—C12A—C13A—C14A	−0.8 (3)	C11B—C12B—C13B—C14B	0.3 (3)
C12A—C13A—C14A—C9A	0.6 (3)	C12B—C13B—C14B—C9B	−0.8 (3)
C10A—C9A—C14A—C13A	−0.5 (3)	C10B—C9B—C14B—C13B	0.5 (3)
C4A—C9A—C14A—C13A	−179.64 (17)	C4B—C9B—C14B—C13B	178.92 (17)
C6A—C5A—C15A—C20A	−54.4 (2)	C6B—C5B—C15B—C16B	−127.1 (2)
C4A—C5A—C15A—C20A	129.1 (2)	C4B—C5B—C15B—C16B	50.9 (3)
C6A—C5A—C15A—C16A	123.8 (2)	C6B—C5B—C15B—C20B	51.4 (3)
C4A—C5A—C15A—C16A	−52.7 (3)	C4B—C5B—C15B—C20B	−130.5 (2)
C20A—C15A—C16A—C17A	−0.5 (3)	C20B—C15B—C16B—C17B	0.8 (3)
C5A—C15A—C16A—C17A	−178.7 (2)	C5B—C15B—C16B—C17B	179.38 (18)
C15A—C16A—C17A—C18A	0.5 (4)	C15B—C16B—C17B—C18B	−0.3 (3)
C16A—C17A—C18A—C19A	−0.5 (4)	C16B—C17B—C18B—C19B	−0.2 (4)
C17A—C18A—C19A—C20A	0.5 (4)	C17B—C18B—C19B—C20B	0.0 (4)
C18A—C19A—C20A—C15A	−0.5 (4)	C18B—C19B—C20B—C15B	0.6 (4)
C16A—C15A—C20A—C19A	0.5 (3)	C16B—C15B—C20B—C19B	−1.0 (3)
C5A—C15A—C20A—C19A	178.7 (2)	C5B—C15B—C20B—C19B	−179.6 (2)

### Table 1 C—H···π interaction geometry (Å, °)

**Table:**

C–H···π	C–H	H···π	C···π	C–H···π
C7A—H7A2···C5A	0.96	2.892	3.780	154.41
C7A—H7A3···C12A	0.96	2.920	3.824	157.38
C8A—H8A1···C16B	0.96	3.014	3.924	158.82
C14A—H14A···C15A	0.93	2.811	3.126	101.09
C16A—H16A···C9A	0.93	2.881	3.167	99.24
C7B—H7B1···C11B	0.96	2.991	3.666	128.53
C8B—H8B1···C12B	0.96	2.943	3.809	150.67
C14B—H14B···C15B	0.93	2.784	3.129	103.11
C16B—H16B···C9B	0.93	2.823	3.138	101.13
